# Data-Driven Network Dynamical Model of Rat Brains During Acute Ictogenesis

**DOI:** 10.3389/fncir.2022.747910

**Published:** 2022-08-10

**Authors:** Victor Hugo Batista Tsukahara, Jordão Natal de Oliveira Júnior, Vitor Bruno de Oliveira Barth, Jasiara Carla de Oliveira, Vinicius Rosa Cota, Carlos Dias Maciel

**Affiliations:** ^1^Signal Processing Laboratory, School of Engineering of São Carlos, Department of Electrical Engineering, University of São Paulo, São Carlos, Brazil; ^2^Laboratory of Neuroengineering and Neuroscience, Department of Electrical Engineering, Federal University of São João Del-Rei, São João Del Rei, Brazil

**Keywords:** network physiology, Local Field Potentials, epilepsy, functional connectivity, Bayesian Networks

## Abstract

Epilepsy is one of the most common neurological disorders worldwide. Recent findings suggest that the brain is a complex system composed of a network of neurons, and seizure is considered an emergent property resulting from its interactions. Based on this perspective, network physiology has emerged as a promising approach to explore how brain areas coordinate, synchronize and integrate their dynamics, both under perfect health and critical illness conditions. Therefore, the objective of this paper is to present an application of (Dynamic) Bayesian Networks (DBN) to model Local Field Potentials (LFP) data on rats induced to epileptic seizures based on the number of arcs found using threshold analytics. Results showed that DBN analysis captured the dynamic nature of brain connectivity across ictogenesis and a significant correlation with neurobiology derived from pioneering studies employing techniques of pharmacological manipulation, lesion, and modern optogenetics. The arcs evaluated under the proposed approach achieved consistent results based on previous literature, in addition to demonstrating robustness regarding functional connectivity analysis. Moreover, it provided fascinating and novel insights, such as discontinuity between forelimb clonus and generalized tonic-clonic seizure (GTCS) dynamics. Thus, DBN coupled with threshold analytics may be an excellent tool for investigating brain circuitry and their dynamical interplay, both in homeostasis and dysfunction conditions.

## 1. Introduction

According to the World Health Organization, approximately fifty million people worldwide suffer from epilepsy, and about 70% of which can live seizure-free using low-cost and effective antiepileptic drugs (WHO, 2019). Temporal lobe epilepsy (TLE), one of its most common forms, is often refractory to pharmacological treatments (Borger et al., 2021; Deng et al., 2021). Furthermore, drug-resistant epileptic patients are often poor candidates for surgical treatment due to the difficulty in identifying the seizure focus (Rincon et al., 2021). From a system-wide standpoint, epilepsy emerges as a hyper synchronization phenomenon based on a modern concept stating that the brain is a complex system and that synchronization is an emergent property resulting from a dynamical coupling of neural oscillators (Moraes et al., 2019; Stojanović et al., 2020). Thereby, hypersynchrony of neural tissues is an essential feature of neurological disorders such as epilepsy and Parkinson's disease (Khaledi-Nasab et al., 2020; Boaretto et al., 2021).

Thus, understanding the dynamic evolution of epilepsy can assist in further elucidating its neurobiological mechanisms (Nelson and Bonner, 2021), which may lead to the development of novel, safer and more efficacious treatments (Moraes et al., 2019). For such a purpose, different analysis have been proposed such as phase Amplitude Coupling (Damborská et al., 2021), Granger Causality (He et al., 2019) or Partial Directed Coherence (Ciaramidaro et al., 2018). There are some limitations in phase Amplitude Coupling, such as a lack of a gold standard set of steps to perform analyzes that may result in misleading interpretations (Seymour et al., 2017). Moreover, it is considered susceptible to sharp-edge artifacts present in some essential electrographic signatures, such as epileptiform spikes which jeopardize its application in epilepsy studies (Kramer et al., 2008). In turn, Granger Causality and Partial Directed Coherence are conflicting linear approaches with the well accepted view that real-world time series are usually nonlinear (Wan and Xu, 2018).

In the current analysis, the relationships and structure of data is understood using a Dynamic Bayesian Network. A Bayesian Network (BN) is a compact representation of statistical dependencies among variables (Neapolitan, 2004; Koller and Friedman, 2009; Bielza and Larrañaga, 2014; Michiels et al., 2021). BNs are probabilistic models defined by a Directed Acyclic Graph (DAG) and conditional probabilities tables (CPT) representing the probabilistic dependence over signals. The Dynamic Bayesian Networks (DBNs) can model signals as BN in successive time slices (Murphy, 2002; Robinson and Hartemink, 2010; Leão et al., 2021). One of its main advantage is that it allows performing probabilistic rationale under uncertainty aiming at associating findings within functional connectivity analysis (Bielza and Larrañaga, 2014; Benjumeda et al., 2021).

In literature, the use of BNs and DBNs in neuroscience is found for multiple purposes (Bielza and Larrañaga, 2014). Eldawlatly et al. (2010) performed a study to find dynamic connectivity between cortical neurons. Smith et al. (2011) inferred a non-linear communication association along regions of the brain; van Esch et al. (2020) used the Bayesian method to evaluate effective connectivity of brain networks aimed to detect the Mozart effect; Sip et al. (2021) developed a data-driven method based on Bayesian Inference to infer seizure propagation patterns in an epileptic brain through intracranial electroencephalography.

Therefore, Bayesian Networks is a remarkable tool to be applied on multidisciplinary analysis, since its graph output is of easy interpretability for specialists of different areas (Chen et al., 2020; Moreira et al., 2021) and it is a good and reliable way to unify algorithmic knowledge from data on specialist knowledge and interpret results when coupled with the multivariate statistical dependence of the model.

The direction of associations between nodes formed in areas of the brain during epileptic seizures is still an unresolved issue (Colmers and Maguire, 2020; Gil et al., 2020; Chowdhury et al., 2021). Tracy et al. (2021) showed that it could change while such seizures occur. Foit et al. (2020) showed that these directions are in fact associated with two critical processes: the generation and expression of seizures and the maintenance of epileptogenic phenomena (Lignani et al., 2020).

This study considers the evolution of brain communication during seizure patterns (or ictogenesis) from a basal state to a generalized tonic-clonic seizure (GTCS) by applying the DBN method to elucidate the link among brain areas for each stage of the process. To clarify communication among brain areas during each time slice as well as elucidating the link between states, such as the relationship of basal and GTCS intervals, rats were exposed to pentylenetetrazole (PTZ) pro-convulsant drugs to induce ictogenesis while their brain local-field activity was recorded and later analyzed through a DBN model.

A detailed model from a set with few instances possesses many attributes, such as time slices and measured variables, which is unexpected. However, it does not mean that the collected dataset lacks essential and helpful information, such as the association trends of main variables. As reported by Koller and Friedman (2009), a strategy to overcome the problem of scarce data and develop a reliable structure of arcs among the nodes of a BN can be the apprenticeship of many high-score structures followed by a consolidation of results. This paper is based on this rationale and suggests applying the threshold analytics proposed by Gross et al. (2019) throughout the dataset of all performed experiments, in addition to highlighting the importance of a sampling approach. The method captures the expected associations among nodes and also achieves better prediction performance than the BNs learned from neighbors thresholds to computed data (Gross et al., 2019), as the method to identify significant arcs proposed by Scutari and Nagarajan (2013).

## 2. Bayesian Networks

A Bayesian Network (BN) is a probabilistic directed acyclic graph (DAG) (Koller and Friedman, 2009; He et al., 2021) represented by nodes as random variables and arcs as the probabilistic relationships. The direction of the arc between two nodes such as Ω and ρ defines a "parent" and "child" node. Ω → ρ means that Ω is the parent of ρ (De Blasi et al., 2021). Mathematically BN is defined as Li et al. (2021):


(1) $$\begin{array}{r} BN=\left(G,\theta \right) \end{array}$$

where *G* = (*X, E*) represents the DAG—the structure of the BN—comprising a set of *n* random variables *X* = {*X*_1_, *X*_2_, ..., *X*_*n*_} as nodes and arcs E. θ = {θ_1_, θ_2_, ..., θ_*n*_} is a set of conditional probability distributions—parameters of the BN (Koller and Friedman, 2009). A data-driven learning in the context of BN involves the apprenticeship of G and θ of a given dataset (de Campos, 2006). Each θ_*i*_ represents a conditional probability distribution *p*(*X*_*i*_|*Pa*_*i*_) in which *Pa*_*i*_ are the parents of *X*_*i*_ in the BN structure (Koller and Friedman, 2009). Using the chain rule from statistics, θ can be used to calculate the joint probability distribution of all specified variables as Lewis and Groth (2020):


(2) $$\begin{array}{r} p\left(X_{1},X_{2},...,X_{n}\right)=\overset{n}{\underset{i=1}{\prod }}p\left(X_{i}|Pa_{i}\right). \end{array}$$

**Figure 1:**
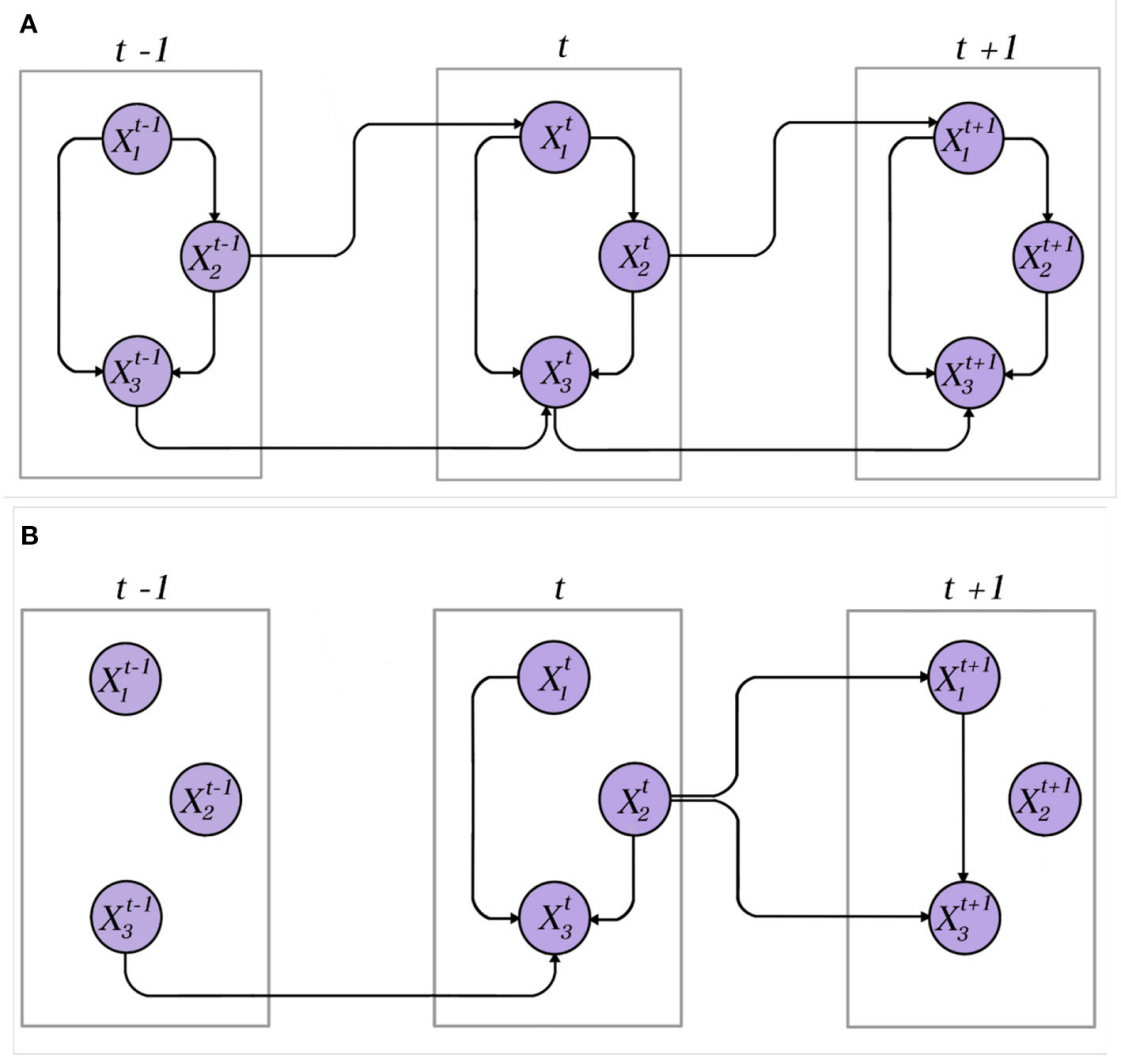
Examples of DBNs. **(A)** A dynamic network in which all variables are represented by stationary time series. The connections between variables within a time slice and in the in-between of time slices are preserved. **(B)** A dynamic network over non-stationary data. The connections between variables and in the in-between of time slices become obsolete and changes over time.

A Dynamic Bayesian Network (DBN) simulates the impact of changes in the BN over time (Li et al., 2021), which means the addition of temporal information to conduct the analysis (Ramos et al., 2021). Figure 1 shows examples of DBNs. This paper simulates the changes during the temporal evolution of rat brains from basal state until the generalized tonic clonic seizure (GTCS), reflecting the changes of communication among brain areas at different timestamps. The DBN method considers that an LFP state (e.g., GTCS) not only depends on variables at a given time *t*_*n*_ (generalized tonic-clonic seizure time) but also in previous time slices, *t*_*n*−*m*_, such as basal state or infusion times for instance.

According to Leão et al. (2021), let *B*_0_ be a prior BN describing the joint distribution between all variables in time slice *t* = 0, *B*_0_ = *P*(**X**[0]). *B*[0:*t*] with *t*∈{1, 2, …, *T*} represents time slices such as *B*[0:*t*] = *P*(**X**[*t*]**X**[0:*t*−1]). The first simplifying assumption is the *m*th-order Markov Property stating that variables in a certain time slice *t* can only be conditionally dependent on variables from up to *m* time slice before *t*. This assumption is simplified into


(3) $$\begin{array}{r} P\left(X\left[0:T\right]\right)=P\left(X\left[0\right]\right)\overset{T}{\underset{t=1}{\prod }}P\left(X\left[t\right]X\left[t-m:t-1\right]\right) \end{array}$$

The second simplifying assumption often performed in DBNs is the stationary assumption stating that, in a DBN following the *m*th-order Markov assumption, transition networks *B*[*t*−*m*:*t*] are the same for all time slices *t*∈{1, ..., *T*}. Both these statements are exhibited in Figure 1, in which both DBNs obey the first-order Markov Property (see Supplementary Material).

## 3. Materials and Methods

This section describes the applied methodology used in this paper. Also, the database used to perform the study, algorithms and their simulated data as well as the computational environment are depicted.

### 3.1. Experimental Protocol

The present experimental procedures with rats are described in better detail in De Oliveira et al. (2018) and its theoretical background is reviewed in Cota et al. (2016) and Cota et al. (2019). Local Field Potential (LFP) originates from the database of the Laboratory of Neuroengineering and Neuroscience (*LINNce*) at the Federal University of São João Del Rei. Male Wistar rats weighing between 250 and 350 grams kept under a light-dark cycle of 12 h (lights on at 7 a.m.) with food and water *ad libitum* were selected from the University's Central Vivarium. All described procedures follow the ethical standards for usage of animals in research and were previously approbated by the institutional committee (protocol 31/2014, CEUA/UFSJ). The signal recording used monopolar electrodes consisting of Teflon-coated stainless-steel wires (#791600, A-M Systems, Sequim, WA, USA). They were placed directly into the right thalamus (TH) and right dorsal hippocampus (HP) of the animal's brains through stereotactic surgery (Cota et al., 2016). The assistance of positioning the electrodes and screws followed Paxino's neuroanatomic atlas and were AP: 2.8 mm, ML: +1.5 mm, DV: 3.3 mm for HP, and AP: 3.0 mm, ML: +2.6 mm, DV: 6,0 mm for TH (Paxinos and Watson, 2013). Additionally, two microsurgical screws (length 4.7 mm, diameter 1.17 mm, Fine Science Tools, Inc., North Vancouver, Canada) were visually implanted in their right hemisphere parietal region for cortical (CX) recording and used the frontal bone as reference. Leads soldered to copper wires were crimped in an RJ-45 jack fixed onto their skull using polymerizing dental acrylic.

Animals were filmed simultaneously in LFP recordings in order to perform a behavioral analysis and assess occurrence and latency to stereotypical behaviors of the chosen models, such as facial automatisms, myoclonic jerk, head clonus, hind and forelimb clonus, generalized tonic-clonic seizure, and others such as rearing and falling, Straub tail. It allowed correlation with LFP data and detection electrophysiological events periods of interest used in this study.

Amplification of signals was performed using a 2,000 V/V gain, filtered from 0.3 to 300 Hz using an A-M Systems (model 3500) pre-amplifier, and then digitized at 1 KSample/s using an A/D converter board (model PCI 6023E, National Instruments) controlled by a built-in LabView virtual instrument developed at *LINNce*. Shielded twisted cables and a Faraday cage were required to eliminate the power grid noise at 60 Hz.

All animals underwent intravenous controlled infusion of convulsant drug pentylenetetrazole (PTZ, Sigma Aldrich, São Paulo, SP—Brazil), an unspecific GABAergic antagonist, at a rate of 1 ml/min and dilution of 10 mg/ml (thus 10 mg/min) as a model of acute ictogenesis and seizure induction. This approach results in a gradual increase of neural excitability and, consequently, gradual recruitment of neural circuitry (Velisek et al., 1992), both expressed behaviorally and electrographically in a correlated manner. Initially, animals display minor seizures, including facial automatisms, strong mastication, myoclonic jerks, forelimb, and head clonus. These are all behaviors directly related to aberrant recruitment of limbic circuitry, including areas such as the amygdala, hippocampus, and thalamus (Eells et al., 2004). It is followed by significant seizures, either with or without a tonic phase, such as generalized myoclonus and generalized tonic-clonic seizures. It results from the involvement of large territories in the forebrain or structures in the midbrain and hindbrain, respectively (Eells et al., 2004). This gradual recruitment of areas and circuits makes the controlled infusion of PTZ an exciting model for screening new drugs or other non-pharmacological treatments. It also investigates neurodynamical processes underlying ictogenesis, such as the case herein.

**Figure 2:**
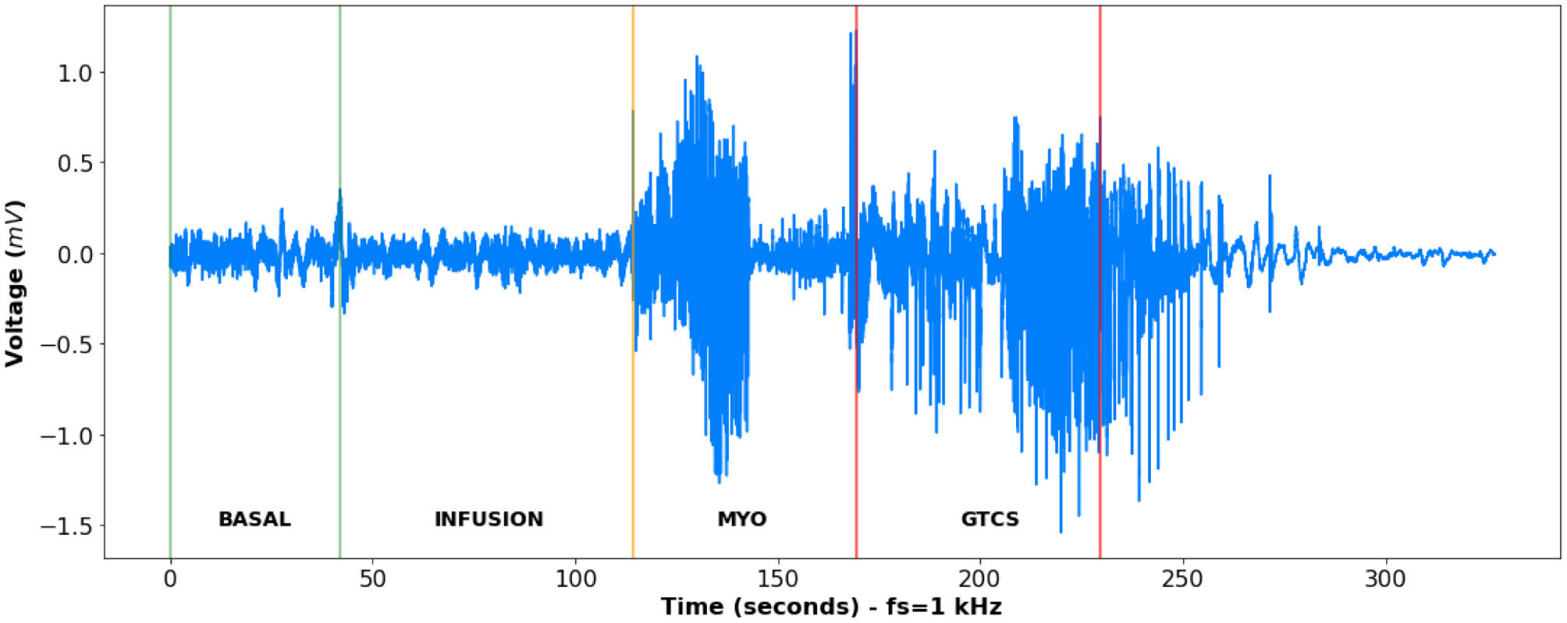
An example of the signal from one of the rats involved in the study. There are four different time slices for it. The first one is the time between the green lines, which indicates resting state, i.e., the basal state of the animal. From the first green line until the orange line, there is an interval of PTZ infusion for epileptic seizure induction. Infusion only ceases when the animal develops generalized tonic clonic seizure. From the orange line until the first red line, there is an interval of myoclonic seizure and from the first red line until the second one representing the period of generalized tonic clonic seizure. After the second red line, it is the interval after GTCS, therefore it is quite evident that this period does not represent a basal state but a refractory period.

The time slices used in this Bayesian Networks analysis were established based on periods of interest of the previously described experimental protocol. The time slices set to apply the algorithm are basal state, infusion, myoclonic seizure (MYO), and generalized tonic-clonic seizure (GTCS)—Figure 2.

### 3.2. Algorithms

**Figure 3:**
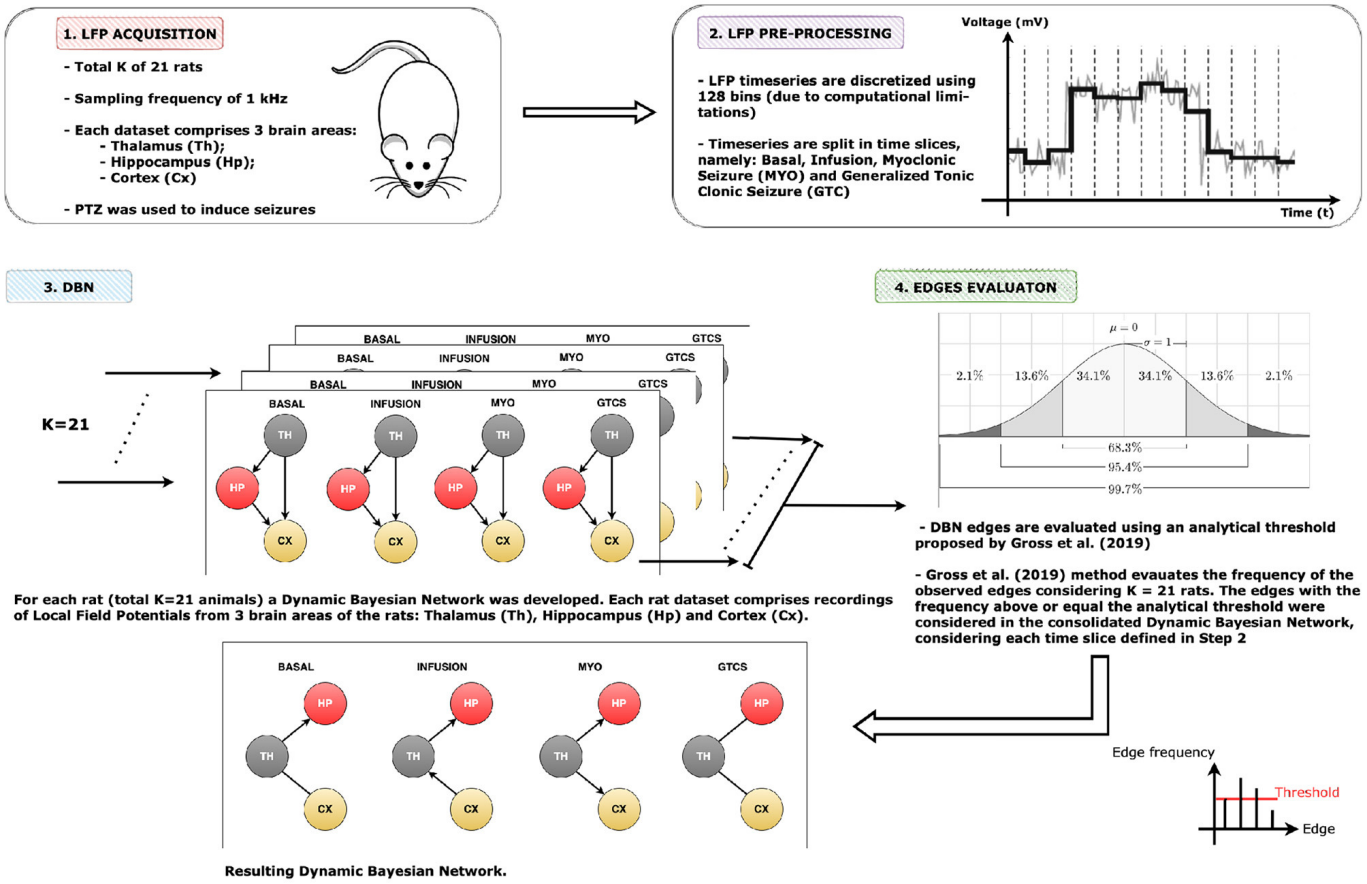
Applied methodology. The initial step is the LFP data acquisition from rats involved in the pre-clinical trial. After discretizing and splitting into time slices, the following point-in-time was depicted: basal state, PTZ infusion, myoclonic seizure (MYO) and generalized tonic clonic seizure (GTCS). The third step involved using the DBN algorithm to observe the functional connectivity among time slices during the temporal evolution of rats, i.e., from basal state until GTCS. The arcs from developed Dynamic Bayesian Networks are evaluated using the analytical threshold model proposed by Gross et al. (2018) described in full detail in Gross et al. (2019).

**Figure 4:**
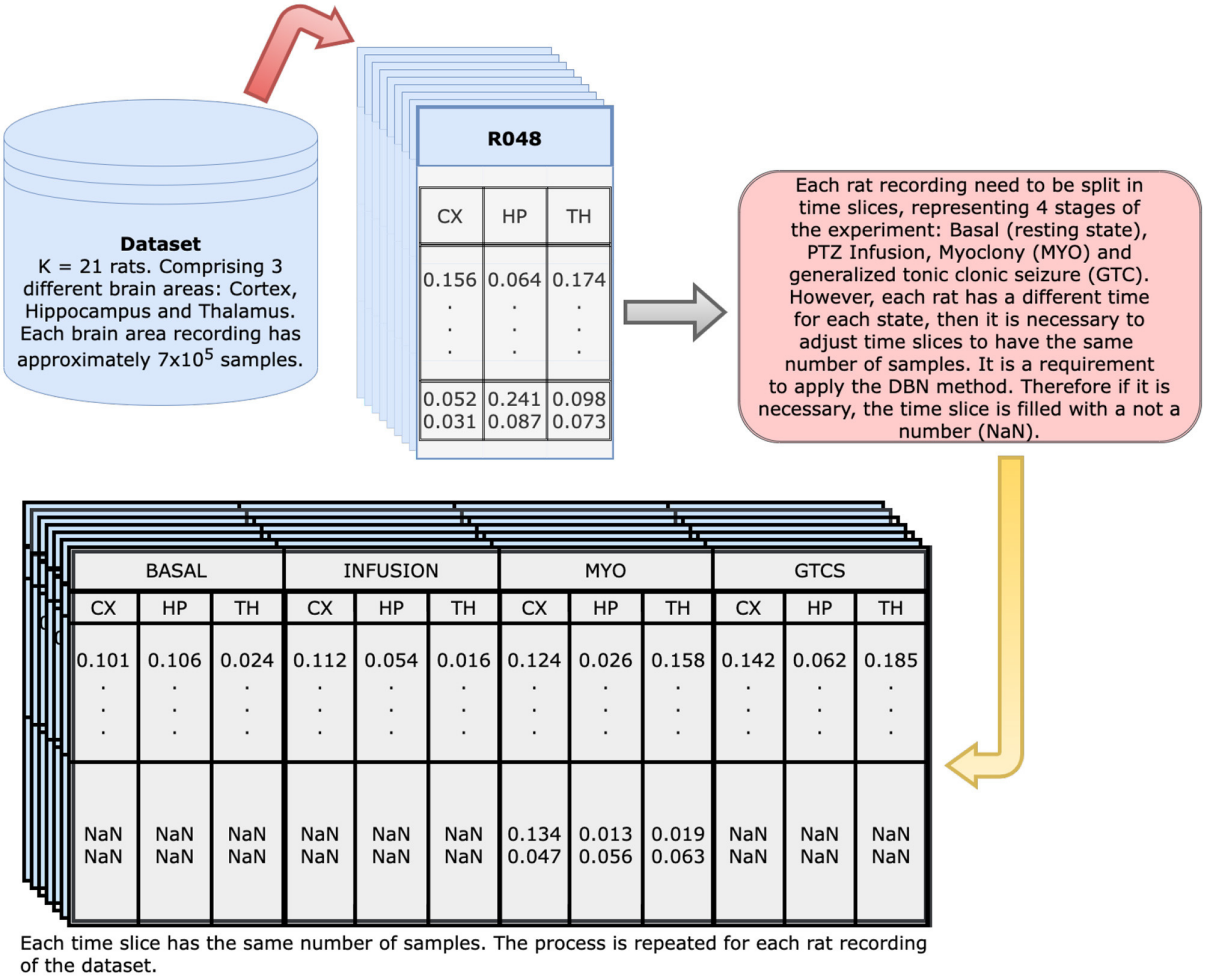
Dynamical Bayesian Network input generation: initial dataset contain 700 thousand observations for each of the three brain regions of each animal; from such, a new dataset is generated with 12 columns representing each of the regions on all of four timestamps (Basal, Infusion, MYO and GTCS). Since all of them have different duration, the resultant table has 12 columns and as many rows as the longest time slice. The remaining columns are filled with NaN to keep the table structure.

Figure 3 presents the applied methodology. Initially, a data frame with three columns (thalamus, hippocampus, and cortex) represents each rat. Afterwards, each of these columns are divided into samples and then regrouped, which resulted in a new data frame with 12 columns: thalamus, hippocampus, and cortex for each time slice, i.e., basal, infusion, MYO, and GTCS (Figure 4). Since each time slice has a different duration, there was pre-processing of all of them using numeric interpolation so that all would have the same size as the longest time slice, resulting in a data frame consisting of 12 columns and *size_of_longest_time-slice* rows. The dependencies among selected variables were based on a completely graphical and non-parametric strategy. The representation of functional connectivity networks among brain areas used a BN structure learned from the discretized dataset.A quantization followed the adaptive bins algorithm was exhibited in Gencaga et al. (2015) using a maximum of 128 bins (7 bits), since it was the maximum amount of bins supported by available computational resources. The Hill Climbing search algorithm from Python *pgmpy* package^1^ was used to learn the DAG from the dataset and the BDeu function (see Appendix) was applied as a scoring method, once the task may be complex or even humanly impossible (Villanueva and Maciel, 2014).

During the experiment, a set of *K* = 21 DAGs was built by running the Hill Climbing search algorithm twenty-one times. Different data used in each of these runs represent a dataset acquired from one of the rats involved in this pre-clinical study. The underlying idea is that there is less uncertainty regarding the arcs induced, even when collecting data from a different animal. Such diversity of structures is due to data acquired from different rats of the same species approximately having exact weights and the Hill Climbing search process itself, once its initialization is always random and local optimizations performed during a run are also non-deterministic. As the stop criterion, for each complete run of the Hill Climbing search algorithm, one million iterations are performed. Afterwards, the set of DAGs was reduced to a single consensus DAG through a process called model-averaging approach. In this reduction, there is a count of the number of times that each of the three possible connections (i.e., “←,” “ → ,” and “absent”) occurred by considering every pair of nodes in the obtained 21 graphs. Only directed arcs having the minimum percentage (f) provided by equation $f=\left(1/3\right)+\sqrt{2/K}$ were accepted. It is the analytical threshold model to evaluate the arcs, proposed by Gross et al. (2018) and described in full detail in Gross et al. (2019); moreover, a specialist analysis was taken into account in the final evaluation of the resulting network, but only the edges resulting from analytical threshold analysis were considered.

The entire algorithm, including the generation of BNs and pre-processing, took about 2–3 min for compiling each rat database, containing three local field potential time series and totaling approximately 700 thousand samples, which was found using a 12 GB RAM and 4-core/4-thread Intel(R) Core(TM) i7-4500U CPU @ 1.80GHz computer. Therefore, the total time spent on processing all databases comprising 21 rats (K = 21) was about 48 min. Among all databases, half the rats belonged to the myoclonic group, and their evolution was recorded from their basal state until the myoclonic seizure. The observation of other rats included a temporal evolution from the basal state until generalized tonic-clonic seizure, i.e., the GTCS group.

## 4. Results

**Figure 5:**
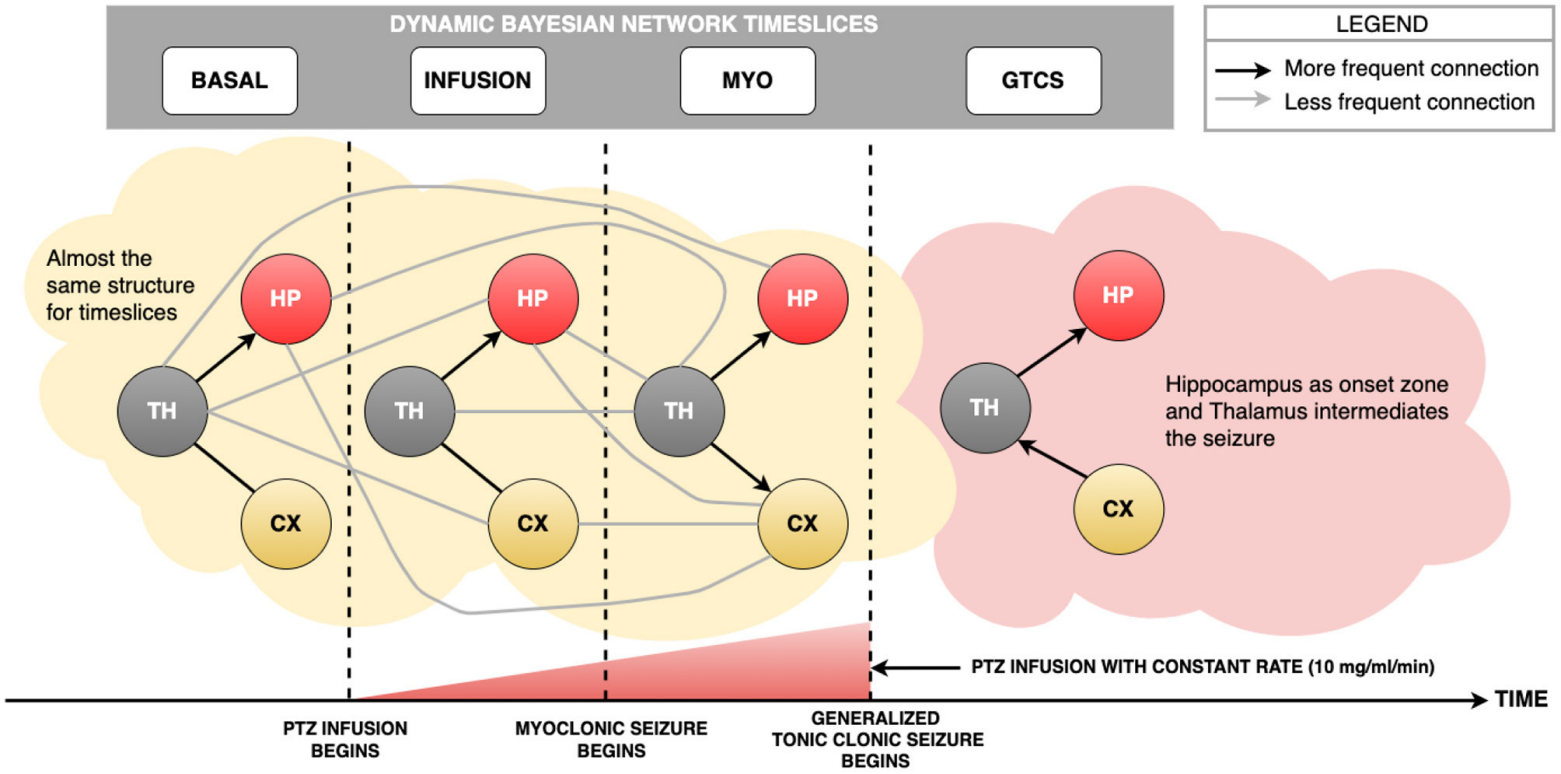
The Dynamic Bayesian Network developed from LFP data set. The black arcs represent the strongest connections provided by analytical threshold model by Gross et al. (2019). The gray arcs represent connections that were not validated by analytical threshold model, but provided by DBN method. From Basal state until MYO time slice, there is a common pattern, i.e., Thalamus distributed information to Hippocampus and Cortex. This behavior is reported in literature, due to fact that knowledge about the role of Thalamus is an important communication lane to distribute information among brain areas. The novelty of the present study is that the same structure of communication is found during the MYO time slice. It was expected a transition structure closer to GTCS, but it did not happen. During GTCS time slice, there was a connection among HP, TH and CX change. It is possible to check that Hippocampus is the probable onset zone, once information reinforces synchronization from Thalamus. Another important path observed and reported in literature is that information from Cortex reverberates in the Thalamus is transmitted to the Hippocampus.

Figures 5, **7** show the use of the LFP database to build the Dynamic Bayesian Network. **Figure 7** represents the Markov Chain evaluation among the employed time slices, and Figure 5 represents the Dynamic Bayesian Network built from rats LFP database. An analysis of arcs using the method suggested by Gross et al. (2019) provided two thresholds: an initial one from the Basal state until the MYO time slice due to further data availability, which provided the value of 0.62 and standard deviation of 0.10, representing the minimum value of 11 arcs. The threshold during GTCS time was 0.71 and standard deviation was 0.16, representing the minimum value of 5 arcs. In Figure 5, it is possible to observe gray edges, but none of them were over the threshold.

Table 1 reports significant arcs, i.e., those having achieved the minimum frequency threshold for each time slice. The table also depicts the number of Dynamic Bayesian Networks in which the connection appeared. There is also a separation between the two groups of rats used in this paper, presenting an overall value used to make a comparison with the analytical threshold value calculated. The exception was only values of GTCS connections if compared with the "GTCS group" column, since the recording of this time slice was performed only on this experimental group.

**Table 1:** The strongest arcs identified in developed Dynamic Bayesian Network.

**Arc ( → )**		**Frequency**		
**From**	**To**	**MYO group**	**GTCS group**	**Global**
Thalamus basal	Cortex basal	7	4	11
Cortex basal	Thalamus basal	7	4	11
Thalamus basal	Hippocampus basal	8	3	11
Thalamus infusion	Cortex infusion	8	3	11
Cortex infusion	Thalamus infusion	7	4	11
Thalamus infusion	Hippocampus infusion	6	6	12
Thalamus MYO	Cortex MYO	8	4	12
Thalamus MYO	Hippocampus MYO	8	7	15
Cortex GTCS	Thalamus GTC	0	7	7
Thalamus GTCS	Hippocampus GTCS	0	6	6

*All of them have the minimum frequency calculated through the analytical threshold model by Gross et al. (2019). Due to the fact that the GTCS time slice is only observed in the GTCS group, the analytical threshold was compared with the frequency observed only for this group. For Basal, Infusion and MYO time slices, the global frequency was used to perform the comparison. Gray edges were not considered in the table, as they have not crossed the analytical threshold (Figure 5)*.

**Figure 6:**
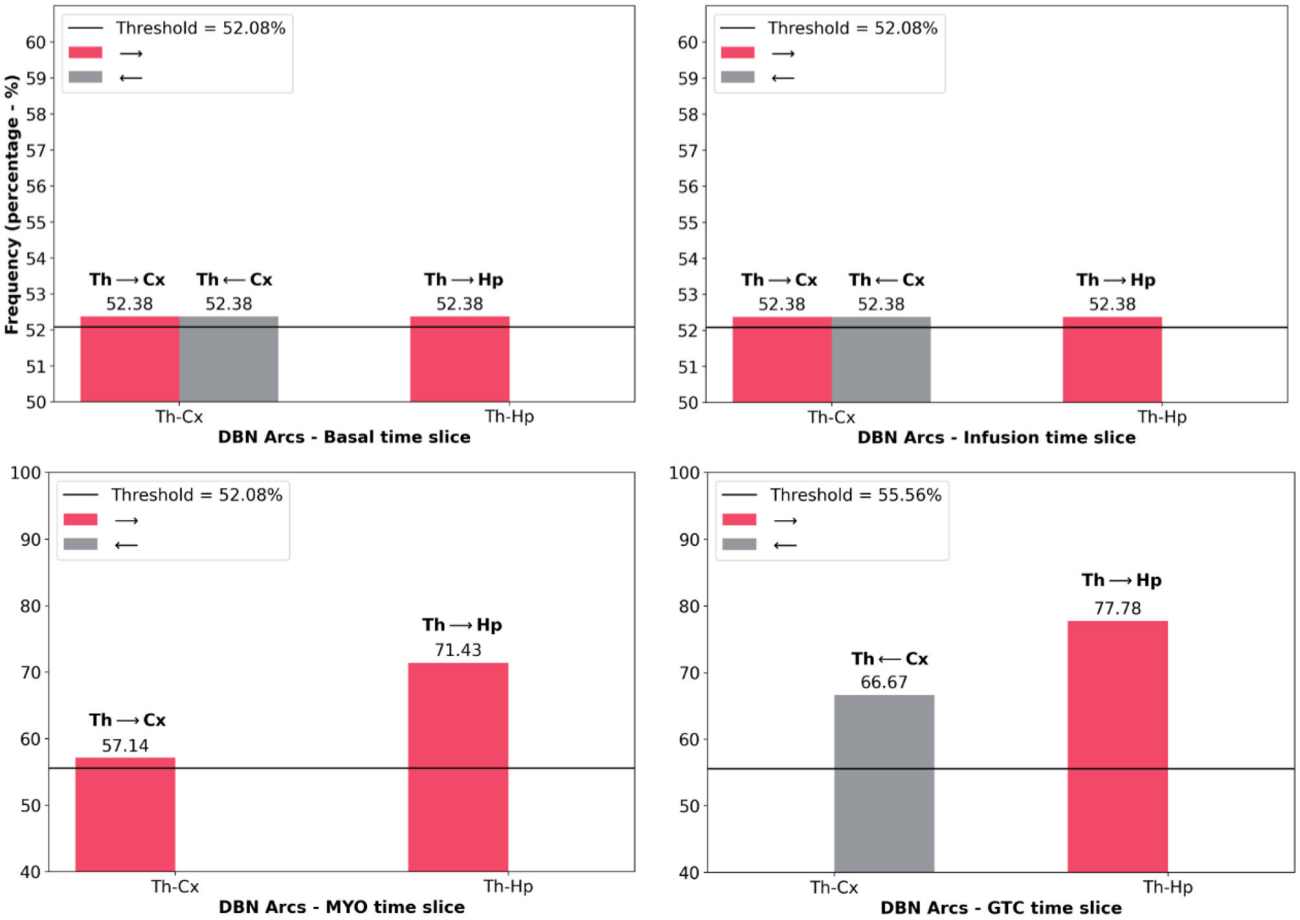
Histograms of the frequency of main arcs from DBN presented in Figure 5. They were separated according to each time slice: Basal, Infusion, MYO and GTCS. Table 1 shows only the arcs that were above the threshold value (black line in histogram) provided by the analytical threshold model. In this Figure, it is also possible to observe that an opposite connection is unchecked at times, such as TH-CX during MYO time slice (TH → CX went through threshold, however, TH←CX did not). The exception is during Basal and Infusion time slices in which it is possible to observe the same frequency for TH-CX arcs.

Figure 6 presents the histogram of significant arcs and their comparison with analytical threshold values. It was possible to observe that there is a single direction from one node to another, such as the case of Thalamus and Hippocampus during the GTCS time slice which was aimed at verifying arc TH → HP. However, TH←HP did not cross the analytical threshold. There is only one exception during Basal and Infusion time slices in which the relationship between Thalamus and Cortex provided the same probability for both directions.

**Figure 7:**
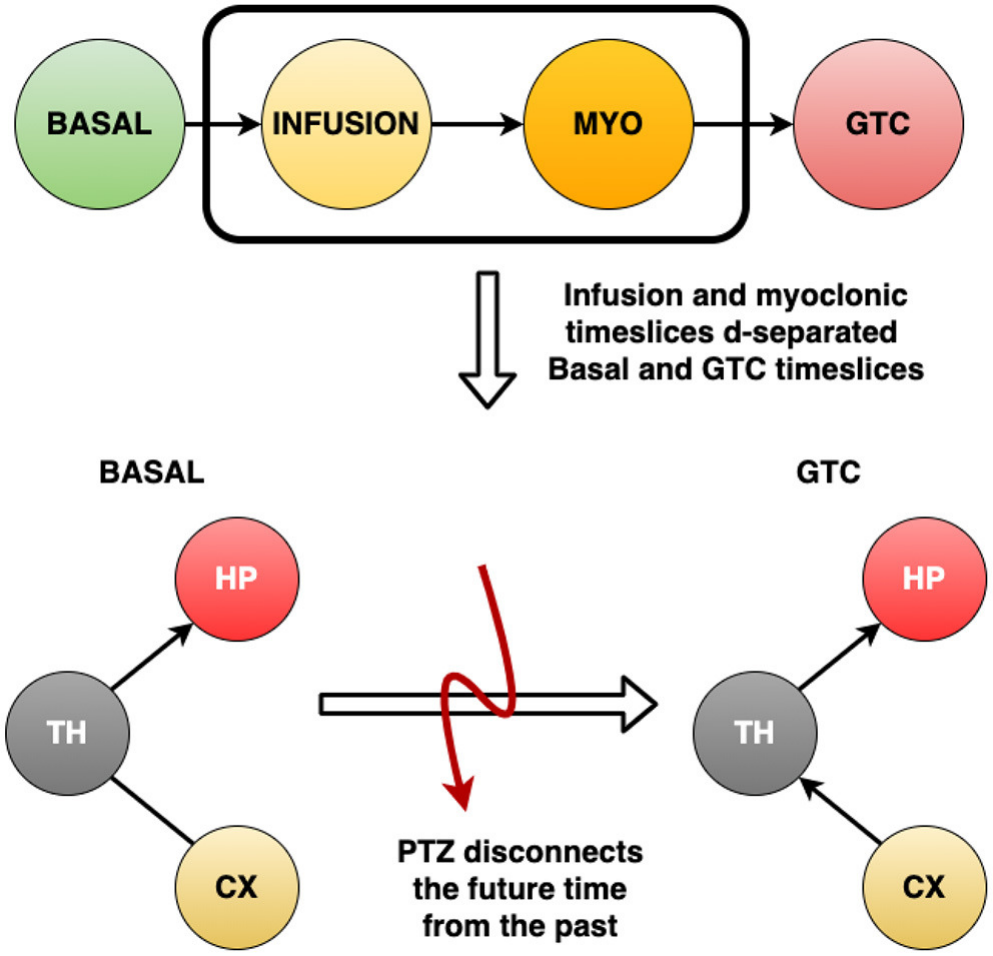
The four time slices used to develop the (Dynamic) Bayesian Network for each rat: Basal, Infusion, MYO and GTCS. After building the networks, it was observed that the Basal interval do not help to explain what happens during GTCS period. Also, it was verified that Infusion and MYO time slices connect them. This means that the infusion of PTZ drug disconnects time slices, which reveals that it is a different process occuring since the beginning of drug administration.

It is possible to observe from Figure 7 that the GTCS time slice does not depend on the Basal time slice. Also, it is suggested that Infusion and MYO time slices connect them. According to Figure 5, the most robust connections patterns were: from Basal until MYO time slice, the interconnection structure remained the same, comprising the Thalamus as the central node connecting to Hippocampus and Cortex, which are in turn independent. The structure changed during the GTCS time slice, and Hippocampus now became the primary node. Cortex connects with Thalamus, which in turn appears linked with Hippocampus. The Dynamic Bayesian Network identified other connections represented in Figure 5, but the analytical threshold has not been validated (gray color).

## 5. Discussion

### 5.1. Temporal Evolution of DAGs Reflecting the Neurodynamics of Ictogenesis

The DBN results found herein have clearly shown distinct connectivity patterns during ictogenesis induced by a controlled infusion of PTZ—Figure 5. Present findings corroborate the dynamic nature of functional neural connectivity along the time course of epileptic phenomena, while also providing novel insights.

A first interesting result is that DAG remains unaltered during the whole PTZ infusion period and it is the same as that in the basal state—Figure 5, i.e., Basal and Infusion time slices. This connectivity pattern observed in both preictal time slices is perfectly understandable and supported by well-understood information flow within neural circuitry in homeostasis. Notably, there is a recognization of the thalamus as the central relay for both incoming sensory information on crossing threshold to multiple primary cortices and also for motor output from the motor cortex—Figure 5, Basal, Infusion, and MYO time slices. Thus, the observed bidirectional link between these nodes is consistent with ongoing sensory and motor function—Figure 5, Basal and Infusion time slices. Additionally, directed arcs from TH to HP are probably related to the communication between the thalamus and hippocampus underlying neural plasticity and acquisition of novel memory traces during wakefulness (Cassel and de Vasconcelos, 2015; Figure 5), Basal, Infusion, and MYO time slices. Such activity is relayed by the thalamus and fed into the hippocampus for future conversion into long-term memories during sleep (Klinzing et al., 2019).

Then, myoclonic seizure starts, and there is a fundamental change in the DAG: the thalamus becomes the primary driver of both the hippocampus and the cortex (notice that the TH to CX arc is now preponderant)—Figure 5, MYO time slice. It is strikingly consistent with the motor expression of partial seizures originating in the limbic system, such as the observed forelimb clonus recorded at this moment, i.e., in which the thalamus assumes the role of a central synchronization hub for both the cortex and the hippocampus (Bertram et al., 1998; Bertram, 2014 for reviews). For this reason, nuclei within the thalamus are in fact the primary targets for neuromodulation strategies in the treatment of epilepsy (Van Der Vlis et al., 2019).

Finally, two essential changes occur when crossing the generalized seizure onset—Figure 5, i.e., GTCS time slice. Initially, the connectivity pattern changes to include a preponderant communication—signal transmission among rats brain areas—from the cortex to the thalamus and thus to the hippocampus. Once more, it is in perfect agreement with previous literature showing the recruitment of vast neocortical territories and communication from these areas to the thalamus and other forebrain structures during secondary generalization (Brodovskaya and Kapur, 2019 for a review). Although a canonical understanding of the importance of the thalamocortical neural circuit, which is a reverberant loop, may imply a bi-directional connection between these two areas, other processes are also crucial for the generation of generalized seizures. These include a unidirectional cortex to thalamus drive through polysynaptic connections involing the basal ganglia (striatum, globus pallidus, and substantia nigra reticulata). Electrode geometry used for cortical recording may also play a unique role herein. Given the larger dimensions of electrodes made out of surgical screws, when compared to microwires used for deep brain recording, signals indeed represent contributions from much larger brain areas. Thus, the aberrant recruitment of vast neocortical territories and their powerful drive onto thalamic nuclei may also be a primary contributor to the preferential direction of the CX toward the TH arc observed in our results. A second significant change after the onset of generalized seizures in the absence of DAG arcs crossing this temporal limit lies in epileptiform activity during partial seizures which bear some neurodynamical correlation with base-level tracings. Meanwhile, generalized tonic-clonic seizures have dynamics of their own that can not correlate with those of other time slices across ictogenesis. The clarity of reasons for this result is yet inexplict, but such an intriguing result may probably have important implications on neuromodulations strategies, particularly upon those involving responsive close-loop systems capable of detecting ongoing seizures.

### 5.2. Graph Evaluation and Analytical Threshold to Identify DBNs Arcs Direction

Figure 7 shows a critical finding: after building the networks, it was found that Basal intervals do not help to explain what happens during the GTCS period. Also, Infusion and MYO time slices connect them. It reveals that the infusion of PTZ drug disconnects the time slices, which means that it is a different ongoing process since the beginning of drug administration. Table 1 substantiated the discussion about the Dynamic Bayesian Network developed herein, suggesting structures in accordance with neuroscience literature. The Dynamic Bayesian Network method brought about many possible connections, as expected. Threshold analytics was essential to analyze their significance, screening the most important arcs, thus enabling a better interpretation of results. Observing Figure 6, the direction of significant arcs is evident, such as Cortex and Thalamus during GTCS time in which CX → TH had a frequency above the threshold (7 against a minimum of 5). However, CX←TH frequency did not cross the threshold (3). According to the methodology, the arcs that were not over threshold frequency represent connections that do not assure their effective existence. Nonetheless, they may be essential tracks for further studies. The most important was an alignment between DBN with threshold analytics and neurobiological phenomena. It is essential to confirm the approach as feasible to investigate epilepsy dynamics. However, according to Bertram (2013), Losi et al. (2019), and Heysieattalab and Sadeghi (2021), there must be more extensive knowledge about the illness dynamics, such as the causal relationship among brain areas.

### 5.3. Other Approaches for Functional Connectivity Analysis and Limitations

In literature, other papers performing Functional Connectivity Analysis are found considering other approaches:

- Tsukahara et al. (2020a) combined Partial Directed Coherence and Mutual Information to study connectivity and transmission rates between brain areas, considering the same dataset used herein and only the Basal and Infusion times under study. However, it only identified connections among all brain areas, but no novel findings regarding ictogenesis were identified.
- Tsukahara et al. (2020b) applied Delayed Mutual Information to identify associations among brain areas considering the lag of communication as regards the same dataset used herein to develop the analysis. The method has not enabled an identification of any novel finding regarding ictogenesis.

As it can be observed, the Information-Theoretic approach, as well a linear approach in the frequency domain, are both commonly used in neuroscience (Ciaramidaro et al., 2018; Gribkova et al., 2018; Varotto et al., 2018), nonetheless there was no novelty regarding ictogenesis phenomena. Some concerns are worthy of consideration in this paper and referenced works: the dataset used to perform analysis. A possible problem that may interfere with results is the acquisition of Local Field Potentials at sampling frequency of 1 kHz. Discretization using the maximum number of bins was required to ensure a better resolution of signals. Endo et al. (2015) performed a similar study as observed in Tsukahara et al. (2020b), however, 32 bins were used to discretize signals sampled at 24 kHz. There is a sharp difference in signals resolution that resulted in different findings. Endo et al. (2015) was able to identify the lag among neurons communications, while Tsukahara et al. (2020b) found no initial lag identification.

Another significant limitation worth being mentioned is the volume of data required to apply the Theoretical Information approach (Endo et al., 2015). The dataset used to perform this analysis is restricted due to availability of rats to perform the study. Within this scenario, a Bayesian approach may be favorable given that initial information provided by a specialist assists in handling smaller datasets, providing results as those observed herein.

Partial Directed Coherence is a linear approach to perform Functional Connectivity analyzes, and real-world problems usually are nonlinear, as it is the case of Local Field Potentials in animal physiology (Phan et al., 2019). Therefore, in addition to the fact that PDC brought about some insights into ictogenesis, it revealed no novelty. Another significant limitation is the requirement of stationarity to apply the method, which can be a problem while studying ictogenesis phenomena.

Despite its limitations, the Dynamic Bayesian Network approach revealed findings in accordance with neuroscience literature and cast light upon some new pieces of knowledge. There are some other questions to be answered considering the subject. However, it is a suggestion for further studies.

Finally, due to data availability to perform the analysis, only three areas were considered in this paper: Thalamus, Hippocampus, and Cortex. Future studies should consider more than three areas aiming at a broad scope to study epileptic seizures. However, only three brain areas assisted to reduce the amount of computational resources to apply DBN analysis and provide findings regarding epileptogenesis.

## 6. Conclusion

The Dynamic Bayesian Network method represents an affordable approach, as there were insights into epilepsy dynamics. It was possible to observe that the infusion of PTZ drug disconnects the timeslices, which means that it has been a different ongoing process since the beginning of drug administration. DBN analysis was very well capable of capturing the dynamic nature of brain connectivity across ictogenesis with significant correlation to neurobiology derived from pioneering studies which employed techniques of pharmacological manipulation, lesion, and modern optogenetics as well (Forcelli, 2017). Additionally, it provided invaluable novel insights, such as the discontinuity between forelimb clonus and GTCS dynamics.

The direction of associations between nodes formed in areas of the brain during epileptic seizures is still an unresolved issue (Colmers and Maguire, 2020; Gil et al., 2020; Chowdhury et al., 2021). This study aimed to address the problem and provide information in agreement with Tracy et al. (2021), showing that basal and infusion time slices present a different pattern of communication than that observed during MYO and GTCS time slices. It is essential to observe that MYO and GTCS time slices present different communication patterns, providing information about the crossing from both states. Furthermore, it suggests evidence of the work of Lignani et al. (2020) from a more focal seizure (MYO) to a tonic-clonic seizure (generalized in GTCS). The study stated that changes in communication direction are associated with two critical processes: the generation and expression of seizure and the epileptogenic phenomenon maintenance.

It was also possible to observe the temporal evolution of variables across time and determine other communications according to the transition from resting-state to the generalized tonic-clonic seizure. Epileptiform activity during partial seizures bears some neurodynamical correlation with base-level tracings. Meanwhile, generalized tonic-clonic seizures have a dynamic of their own that cannot correlate with those of other time slices across ictogenesis. The clarity of reasons for such result is yet unclear. Nevertheless, such intriguing result might have important implications on neuromodulation strategies, particularly those involving responsive close-loop systems capable of detecting ongoing seizures.

For these reasons, DBN might be an excellent tool for investigating brain circuitry and its dynamical interplay in both homeostasis and dysfunction. Analytical threshold results supported all this discussion due to allowing an evaluation of the arc's significance and identifying the connections observed through the developed DBN.

Computationally, the applied methodology demonstrated to be an appropriate alternative. Each rat data frame spent about 3 min running and provided a suggested DBN model. All databases were run in about 50 min, which is quite fast, mainly on account of the fact that the DBN method is an NP-hard problem. This study was carried out using a personal computer without any adaptations, which is also relevant as it contributes to the results reproducibility. Thus, the algorithms presented two features that support its availability to perform functional connectivity analysis: good computational time of processing and reproducibility.

Therefore, the approach demonstrated that it is feasible to investigate epilepsy dynamics, once important insights reported in literature were identified, in addition to new findings. As suggestion for further studies, there is still the need for more knowledge about the illness dynamics, such as using more brain areas to increase the scope of observation of the epileptogenic dynamics. Also, the use of Local Field Potentials might be applied at more sampling frequency to make signal representation more precise, thus increasing the applied methodology's quality. Finally, using the proposed methodology to study other types of brain disorders, like Parkinson's disease, seems prominent as further research.

## Data Availability Statement

The raw data supporting the conclusions of this article will be made available by the authors upon request, without undue reservation. Requests to access these datasets should be directed to vrcota@ufsj.edu.

## Ethics Statement

The animal study was reviewed and approved by Ethical Review Board of the Federal University of São João Del-Rei (Ethics Committee Protocol 31/2014).

## Author Contributions

VB, JNO, and VO designed, drafted and revised the manuscript. JCO designed and performed animal experiments and data acquisition. VR supervised data acquisition and experiments, designed, drafted and revised the manuscript. CM designed, drafted and revised the manuscript. No undisclosed groups involved in this study. All authors have seen and approved the submitted version of the paper and accept responsibility for its content.

## Funding

This work was partialy supported by following agencies: FAPEMIG APQ 02485-15, CAPES-Finance Code 001, FAPESP 2014/50851-0, CNPq 465755/2014-3, and BPE Fapesp 2018/19150-6.

## Conflict of Interest

The authors declare that the research was conducted in the absence of any commercial or financial relationships that could be construed as a potential conflict of interest.

## Publisher's Note

All claims expressed in this article are solely those of the authors and do not necessarily represent those of their affiliated organizations, or those of the publisher, the editors and the reviewers. Any product that may be evaluated in this article, or claim that may be made by its manufacturer, is not guaranteed or endorsed by the publisher.

## Footnotes

^1^ http://pgmpy.org/index.html

## References

[B1] Benjumeda M. Tan Y.-,l. González Otárula K. A. Chandramohan D. Chang E. F. Hall J. A.. (2021). Patient specific prediction of temporal lobe epilepsy surgical outcomes. Epilepsia 62, 2113–2122. 10.1111/epi.17002 34275140

[B2] Bertram E. H.. (2013). Neuronal circuits in epilepsy: do they matter? Exp. Neurol. 244, 67–74. 10.1016/j.expneurol.2012.01.028 22342991 PMC4266595

[B3] Bertram E. H.. (2014). Extratemporal lobe circuits in temporal lobe epilepsy. Epilepsy Behav. 38, 13–18. 10.1016/j.yebeh.2014.07.012 25238899

[B4] Bertram E. H. Mangan P. Fountain N. Rempe D.. (1998). Functional anatomy of limbic epilepsy: a proposal for central synchronization of a diffusely hyperexcitable network. Epilepsy Res. 32, 194–205. 10.1016/S0920-1211(98)00051-5 9761320

[B5] Bielza C. Larrañaga P. (2014). Bayesian networks in neuroscience: a survey. Front. Comput. Neurosci. 8, 131. 10.3389/fncom.2014.00131 PMC4199264 25360109

[B6] Boaretto B. Manchein C. Prado T. Lopes S. (2021). The role of individual neuron ion conductances in the synchronization processes of neuron networks. Neural Networks 137, 97–105. 10.1016/j.neunet.2021.01.019 33578080

[B7] Borger V. Hamed M. Taube J. Aydin G. Ilic I. Schneider M.. (2021). Resective temporal lobe surgery in refractory temporal lobe epilepsy: prognostic factors of postoperative seizure outcome. J. Neurosurg. 1, 1–10. 10.3171/2020.7.JNS20284 42157441

[B8] Brodovskaya A. Kapur J. (2019). Circuits generating secondarily generalized seizures. Epilepsy Behav. 101, 106474. 10.1016/j.yebeh.2019.106474 PMC6944760 31431400

[B9] Cassel J.-C. de Vasconcelos A. P. (2015). Importance of the ventral midline thalamus in driving hippocampal functions. Prog Brain Res. 219, 145–161. 10.1016/bs.pbr.2015.03.005 26072238

[B10] Chen J. Dai X. Yuan Q. Lu C. Huang H. (2020). “Towards interpretable clinical diagnosis with bayesian network ensembles stacked on entity-aware cnns,” in Proceedings of the 58th Annual Meeting of the Association for Computational Linguistics (Washington, D.C.), 3143–3153.

[B11] Chowdhury F. A. Silva R. Whatley B. Walker M. C. (2021). Localisation in focal epilepsy: a practical guide. Pract. Neurol. 21, 481–491. 10.1136/practneurol-2019-002341 34404748

[B12] Ciaramidaro A. Toppi J. Casper C. Freitag C. Siniatchkin M. Astolfi L. (2018). Multiple-brain connectivity during third party punishment: an eeg hyperscanning study. Scientific Rep. 8, 6822. 10.1038/s41598-018-24416-w PMC5931604 29717203

[B13] Colmers P. L. Maguire J. (2020). Network dysfunction in comorbid psychiatric illnesses and epilepsy. Epilepsy Curr. 20, 205–210. 10.1177/1535759720934787 32628514 PMC7427163

[B14] Cota V. Marcela Bacellar Drabowski B. Carla de Oliveira J. Moraes M. (2016). The epileptic amygdala: toward the development of a neural prosthesis by temporally coded electrical stimulation. J. Neurosci. Res. 94, 463–485. 10.1002/jnr.23741 27091311

[B15] Cota V. R. de Oliveira J. C. Damázio L. C. M. Moraes M. F. D. (2019). Nonperiodic stimulation for the treatment of refractory epilepsy: applications, mechanisms, and novel insights. Epilepsy Behav. 21(Pt B), 106609. 10.1016/j.yebeh.2019.106609 31704250

[B16] Damborská A. Lamoš M. Brunet D. Vulliemoz S. Bočková M. Deutschová B.. (2021). Resting-state phase-amplitude coupling between the human subthalamic nucleus and cortical activity: a simultaneous intracranial and scalp eeg study. Brain Topogr. 34, 272–282. 10.1007/s10548-021-00822-8 33515171

[B17] De Blasi R. A. Campagna G. Finazzi S. (2021). A dynamic bayesian network model for predicting organ failure associations without predefining outcomes. PLoS ONE 16, e0250787. 10.1371/journal.pone.0250787 33909682 PMC8081190

[B18] de Campos L. M.. (2006). A scoring function for learning bayesian networks based on mutual information and conditional independence tests. J. Mach. Learn. Res. 7, 2149–2187.

[B19] De Oliveira J. Maciel R. Moraes M. Cota V. R. (2018). Asynchronous, bilateral, and biphasic temporally unstructured electrical stimulation of amygdalae enhances the suppression of pentylenetetrazole-induced seizures in rats. Epilepsy Res. 146, 1–8. 10.1016/j.eplepsyres.2018.07.009 30053674

[B20] Deng N. Hu J. Hong Y. Ding Y. Xiong Y. Wu Z.. (2021). Indoleamine-2, 3-dioxygenase 1 deficiency suppresses seizures in epilepsy. Front. Cell Neurosci. 15, 28. 10.3389/fncel.2021.638854 PMC7935521 33679331

[B21] Eells J. Clough R. Browning R. Jobe P. (2004). Comparative fos immunoreactivity in the brain after forebrain, brainstem, or combined seizures induced by electroshock, pentylenetetrazol, focally induced and audiogenic seizures in rats. Neuroscience 123, 279–292. 10.1016/j.neuroscience.2003.08.015 14667462

[B22] Eldawlatly S. Zhou Y. Jin R. Oweiss K. G. (2010). On the use of dynamic bayesian networks in reconstructing functional neuronal networks from spike train ensembles. Neural Comput. 22, 158–189. 10.1162/neco.2009.11-08-900 19852619 PMC2794930

[B23] Endo W. Santos F. P. Simpson D. Maciel C. D. Newland P. L. (2015). Delayed mutual information infers patterns of synaptic connectivity in a proprioceptive neural network. J. Comput. Neurosci. 38, 427–438. 10.1007/s10827-015-0548-6 25643986

[B24] Foit N. A. Bernasconi A. Bernasconi N. (2020). Functional networks in epilepsy presurgical evaluation. Neurosurg. Clin. 31, 395–405. 10.1016/j.nec.2020.03.004 32475488

[B25] Forcelli P. A.. (2017). Applications of optogenetic and chemogenetic methods to seizure circuits: where to go next? J. Neurosci. Res. 95, 2345–2356. 10.1002/jnr.24135 28791729 PMC5647238

[B26] Gencaga D. Knuth K. H. Rossow W. B. (2015). A recipe for the estimation of information flow in a dynamical system. Entropy 17, 438–470. 10.3390/e17010438

[B27] Gil F. Padilla N. Soria-Pastor S. Setoain X. Boget T. Rumiá J.. (2020). Beyond the epileptic focus: functional epileptic networks in focal epilepsy. Cereb. Cortex 30, 2338–2357. 10.1093/cercor/bhz243 31867595

[B28] Gribkova E. D. Ibrahim B. A. Llano D. A. (2018). A novel mutual information estimator to measure spike train correlations in a model thalamocortical network. J. Neurophysiol. 120, 2730–2744. 10.1152/jn.00012.2018 30183459 PMC6337027

[B29] Gross T. J. Araújo R. B. Vale F. A. C. Bessani M. Maciel C. D. (2018). Dependence between cognitive impairment and metabolic syndrome applied to a brazilian elderly dataset. Artif. Intell. Med. 90, 53–60. 10.1016/j.artmed.2018.07.003 30076067

[B30] Gross T. J. Bessani M. Junior W. D. Araujo R. B. Vale F. A. C. Maciel C. D. (2019). An analytical threshold for combining bayesian networks. Knowl. Based Syst. 175, 36–49. 10.1016/j.knosys.2019.03.014

[B31] He B. Astolfi L. Valdés-Sosa P. A. Marinazzo D. Palva S. O. Bénar C.-G.. (2019). Electrophysiological brain connectivity: theory and implementation. IEEE Trans. Biomed. Eng. 66, 2115–2137. 10.1109/TBME.2019.2913928 31071012 PMC6834897

[B32] He C. Yu H. Gu S. Zhang W. (2021). A multi-granularity information-based method for learning high-dimensional bayesian network structures. Cogn. Comput. 13, 1–13. 10.1007/s12559-021-09891-0

[B33] Heysieattalab S. Sadeghi L. (2021). Dynamic structural neuroplasticity during and after epileptogenesis in a pilocarpine rat model of epilepsy. Acta Epileptol. 3, 1–9. 10.1186/s42494-020-00037-7

[B34] Khaledi-Nasab A. Kromer J. A. Tass P. A. (2020). Long-lasting desynchronization of plastic neural networks by random reset stimulation. Front. Physiol. 11, 1843. 10.3389/fphys.2020.622620 PMC7893102 33613303

[B35] Klinzing J. G. Niethard N. Born J. (2019). Mechanisms of systems memory consolidation during sleep. Nat. Neurosci. 22, 1598–1610. 10.1038/s41593-019-0467-3 31451802

[B36] Koller D. Friedman N. (2009). Probabilistic Graphical Models: Principles and Techniques. Cambridge, MA: MIT Press.

[B37] Kramer M. A. Tort A. B. Kopell N. J. (2008). Sharp edge artifacts and spurious coupling in eeg frequency comodulation measures. J. Neurosci. Methods 170, 352–357. 10.1016/j.jneumeth.2008.01.020 18328571

[B38] Leã T. Madeira S. C. Gromicho M. de Carvalho M. Carvalho A. M. (2021). Learning dynamic bayesian networks from time-dependent and time-independent data: unraveling disease progression in amyotrophic lateral sclerosis. J. Biomed. Inform. 117, 103730. 10.1016/j.jbi.2021.103730 33737206

[B39] Lewis A. D. Groth K. M. (2020). A dynamic bayesian network structure for joint diagnostics and prognostics of complex engineering systems. Algorithms 13, 64. 10.3390/a13030064

[B40] Li X. Zhang Y. Li Y. Zhan Y. Yang L. (2021). Health state prediction and performance evaluation of belt conveyor based on dynamic bayesian network in underground mining. Shock Vibrat. 2021, 6699611. 10.1155/2021/6699611

[B41] Lignani G. Baldelli P. Marra V. (2020). Homeostatic plasticity in epilepsy. Front. Cell Neurosci. 14, 197. 10.3389/fncel.2020.00197 PMC7333442 32676011

[B42] Losi G. Gomez-Gonzalo M. Zonta M. Chiavegato A. Carmignoto G. (2019). Cellular and molecular mechanisms of new onset seizure generation. Aging Clin. Exp. Res. 33, 1713–1716. 10.1007/s40520-019-01396-z 31732960

[B43] Michiels M. Larra naga P. Bielza C. (2021). Bayesuites: An open web framework for massive bayesian networks focused on neuroscience. Neurocomputing 428, 166–181. 10.1016/j.neucom.2020.11.066

[B44] Moraes M. F. D. de Castro Medeiros D. Mourao F. A. G. Cancado S. A. V. Cota V. R. (2019). Epilepsy as a dynamical system, a most needed paradigm shift in epileptology. Epilepsy Behav. 121, 106838. 10.1016/j.yebeh.2019.106838 31859231

[B45] Moreira C. Chou Y.-L. Velmurugan M. Ouyang C. Sindhgatta R. Bruza P. (2021). Linda-bn: an interpretable probabilistic approach for demystifying black-box predictive models. Decis. Support Syst. 150, 113561. 10.1016/j.dss.2021.113561

[B46] Murphy K. P.. (2002). Dynamic bayesian networks: representation, inference and learning (Master's thesis). University of California, Berkeley Berkeley, CA.

[B47] Neapolitan R. E.. (2004). Learning Bayesian Networks, Vol. 38. Upper Saddle River, NJ: Pearson Prentice Hall.

[B48] Nelson C. J. Bonner S. (2021). Neuronal graphs: A graph theory primer for microscopic, functional networks of neurons recorded by calcium imaging. Front. Neural Circ. 15, 38. 10.3389/fncir.2021.662882 PMC8222695 34177469

[B49] Paxinos G. Watson C. (2013). The Rat Brain in Stereotaxic Coordinates, 7th Edn. Elsevier. 10.1016/0165-0270(80)90021-7 6110810

[B50] Phan T. D. Wachter J. A. Solomon E. A. Kahana M. J. (2019). Multivariate stochastic volatility modeling of neural data. eLife 8, e42950. 10.7554/eLife.42950.026 31368892 PMC6697415

[B51] Ramos D. Ramirez-Hereza P. Toledano D. T. Gonzalez-Rodriguez J. Ariza-Velazquez A. Solis-Tovar D.. (2021). Dynamic bayesian networks for temporal prediction of chemical radioisotope levels in nuclear power plant reactors. Chemometr. Intell. Lab. Syst. 214, 104327. 10.1016/j.chemolab.2021.104327

[B52] Rincon N. Barr D. Velez-Ruiz N. (2021). Neuromodulation in drug resistant epilepsy. Aging Dis. 12, 1070–1080. 10.14336/AD.2021.0211 34221550 PMC8219496

[B53] Robinson J. W. Hartemink A. J. (2010). Learning non-stationary dynamic bayesian networks. J. Mach. Learn. Res. 11, 3647–3680.31544173

[B54] Scutari M. Nagarajan R. (2013). Identifying significant edges in graphical models of molecular networks. Artif. Intell. Med. 57, 207–217. 10.1016/j.artmed.2012.12.006 23395009 PMC4070079

[B55] Seymour R. A. Rippon G. Kessler K. (2017). The detection of phase amplitude coupling during sensory processing. Front. Neurosci. 11, 487. 10.3389/fnins.2017.00487 PMC5585190 28919850

[B56] Sip V. Hashemi M. Vattikonda A. N. Woodman M. M. Wang H. Scholly J.. (2021). Data-driven method to infer the seizure propagation patterns in an epileptic brain from intracranial electroencephalography. PLoS Comput. Biol. 17, e1008689. 10.1371/journal.pcbi.1008689 PMC7920393 33596194

[B57] Smith S. M. Miller K. L. Salimi-Khorshidi G. Webster M. Beckmann C. F. Nichols T. E.. (2011). Network modelling methods for fmri. Neuroimage 54, 875–891. 10.1016/j.neuroimage.2010.08.063 20817103

[B58] Stojanović O. Kuhlmann L. Pipa G. (2020). Predicting epileptic seizures using nonnegative matrix factorization. PLoS ONE 15, e0228025. 10.1371/journal.pone.0228025 32023272 PMC7001919

[B59] Tracy J. I. Chaudhary K. Modi S. Crow A. Kumar A. Weinstein D.. (2021). Computational support, not primacy, distinguishes compensatory memory reorganization in epilepsy. Brain Commun. 3, fcab025. 10.1093/braincomms/fcab025 PMC8244645 34222865

[B60] Tsukahara V. H. Jeronymo P. V. Oliveira J. C. d Cota V. R. Maciel C. D. (2020a). “PDC-MI method for eeg functional conectivity analysis,” in International Joint Conference on Biomedical Engineering Systems and Technologies (Springer), 304–328. 10.1007/978-3-030-72379-8_15

[B61] Tsukahara V. H. B. Jeronymo P. V. B. de Oliveira J. C. Cota V. R. Maciel C. D. (2020b). “Delayed mutual information to develop functional analysis on epileptic signals,” in BIOSIGNALS, 89–97.

[B62] Van Der Vlis T. A. B. Schijns O. E. Schaper F. L. Hoogland G. Kubben P. Wagner L.. (2019). Deep brain stimulation of the anterior nucleus of the thalamus for drug-resistant epilepsy. Neurosurg Rev. 42, 287–296. 10.1007/s10143-017-0941-x 29306976 PMC6502776

[B63] van Esch R. J. Shi S. Bernas A. Zinger S. Aldenkamp A. P. Van den Hof P. M. (2020). A bayesian method for inference of effective connectivity in brain networks for detecting the mozart effect. Comput. Biol. Med. 127, 104055. 10.1016/j.compbiomed.2020.104055 33157484

[B64] Varotto G. Franceschetti S. Caputo D. Visani E. Canafoglia L. Freri E.. (2018). Network characteristics in benign epilepsy with centro-temporal spikes patients indicating defective connectivity during spindle sleep: a partial directed coherence study of eeg signals. Clin. Neurophysiol. 129, 2372–2379. 10.1016/j.clinph.2018.09.008 30268930

[B65] Velisek L. Kubova H. Pohl M. Stankova L. Mareš P. Schickerova R. (1992). Pentylenetetrazol-induced seizures in rats: an ontogenetic study. Naunyn Schmiedebergs Arch. Pharmacol. 346, 588–591. 10.1007/BF00169017 1470230

[B66] Villanueva E. Maciel C. D. (2014). Efficient methods for learning bayesian network super-structures. Neurocomputing 123, 3–12. 10.1016/j.neucom.2012.10.035

[B67] Wan X. Xu L. (2018). A study for multiscale information transfer measures based on conditional mutual information. PLoS ONE 13, e0208423. 10.1371/journal.pone.0208423 30521578 PMC6283631

[B68] WHO (2019). Epilepsy: A Public Health Imperative. Geneva: World Health Organization.

