# Supplementary material for: Data-Driven Network Dynamical Model of Rat Brains During Acute Ictogenesis
Source: Front Neural Circuits. 2022 Aug 10;16:747910. doi: 10.3389/fncir.2022.747910 (PMC9399918; doi:10.3389/fncir.2022.747910)
Supplement: Supplementary file 1 [file Data_Sheet_1.pdf]

Supplementary Material

1 APPENDIX: BAYESIAN NETWORKS

A Directed Graph (DG) is a pair (V, E) in which V is a finite and non-empty set of vertices and E is a set of distinct pairs (X, Y) in which $X \to Y$ are both in V and we say that there is an edge between X and Y. In this case, X and Y are said to be adjacent, and X is a parent of Y and Y is a descendent of X. In the set $\{X_1, X_2, \ldots, X_n\}$ given the rule of association ($X_{i-1} \to X_i, i \geq 2$), the nodes $\{X_2, X_3, \ldots X_n\}$ are called interior nodes. The sub-path from X_i to X_j in $\{X_1, X_2, \ldots, X_n\}$ is the path $\{X_i, X_{i+1}, \ldots, X_j\}$ in which $1 \leq i < j \leq n$. A directed cycle is a path from a node to itself. A Directed Acyclic Graph (DAG) G is a DG in which there are no directed cycles (Neapolitan et al., 2004).

Definition 1.1. Suppose we have a joint probability distribution P of the random variables in some set V and a DAG $G = (V, E)$. We say that (G, P) satisfies the Markov condition if for each variable $X \in V$, X is independent conditional to the set of all its non-descendents ND_X given the set of all its parents PA_X, i.e.

$$X \perp\!\!\!\perp \mathrm{ND}_X | \mathrm{PA}_X \tag{S1}$$

THEOREM 1.2. *If (G, P) satisfies the Markov condition, then P is equal to the product of its conditional distributions of all nodes given values of their parents, whenever these conditional distributions exist, i.e., if the set of nodes $\{X_1, X_2, \ldots, X_n\}$ is ordered in ancestral ordering (if X_k is a descendent of X_j, it appears later in the ordering) we can write*

$$P(x_1, x_2, \ldots, x_n) = P(x_n|\mathrm{pa}_n)P(x_{n-1}|\mathrm{pa}_{n-1})\ldots P(x_1|\mathrm{pa}_1) \tag{S2}$$

in which the set of values $\{x_1, x_2, \ldots, x_n\}$ represents states of the variables $\{X_1, X_2, \ldots, X_n\}$ and pa_i are the subsets of these values containing the values of X_i's parents.

Let again P be the joint distribution function of random variables in the set of vertices V in a graph G. If (G, P) obeys the Markov condition, it is said that (G, P) is a Bayesian Network (BN). For historical and algorithmic reasons, it is more common to refer to a BN as the pair (G, Θ) in which $G = (V, E)$ is its corresponding DAG and $\Theta = \{\theta_1, \theta_2, \ldots, \theta_n\}$ represents a set of conditional probability distributions encoded in the DAG G. For any BN with joint distribution P and vertices V on the set of random variables $\{X_1, X_2, \ldots, X_n\}$, the factorization from Equation S2 can be resumed as

$$P = \prod_{x \in \{X\}} p(x|\mathrm{pa}_x) \tag{S3}$$

Bayesian Networks are used in several areas of knowledge-based systems, such as speech recognition (Nasereddin and Omari, 2017; Zweig, 2003; Zweig and Russell, 1998), climate studies (Lee et al., 2020; Moe et al., 2016), population studies (Cai, 2017; McCann et al., 2006) and machine learning (Jiang et al., 2019; Chaturvedi et al., 2018). It has the advantage of encoding information about the interaction inside

multivariate systems in the dependencies represented by its edges. There are, however, a series of complex problems when using the BN approaching in real-world data.

Given a dataset with variables $\{X_1, X_2, \ldots, X_n\}$, the more likely structure G is found by a *learning algorithm*. The number of possible DAG's with n vertices grows in a super-exponential way, given by the recurrence relationship (Robinson, 1977):

$$G(n) = \sum_{k=1}^{n} (-1)^{k+1} \binom{n}{k} 2^{k(n-k)} G(n-k) \tag{S4}$$

There are also lower and upper bound (Stanley, 1973): $G_{lower} = 2^{\frac{n(n-1)}{2}}$ and $G_{upper} = n!2^{\frac{n(n-1)}{2}}$. This explosive growth (superexponential) of the number of possible DAGs given the number of vertices indicates that it is not possible to do an intensive search. There are several approaches to find a reasonably good fit for the data. These algorithms are based on scoring the edges on the possible DAGs and using some kind of evolutionary algorithm, such as Particle Swarm Optimisation (Kouziokas, 2020), Hill-Climbing Search (Tsamardinos et al., 2006) and Genetic Algorithms (Contaldi et al., 2019; Larranaga et al., 1996). A learning algorithm has, therefore two components: a scoring metric and a search procedure. It raises the question of how to define a score-based system to choose from the super-exponential DAG space using any algorithm to search.

A score-function for learning BN models from data was developed by Cooper and Herskovits (1992) using four assumptions: the database variables are discrete, cases occur independently, given a belief-network model, there are no cases that have variables with missing values before we observe database D, we are indifferent regarding the numerical probabilities to place on belief-network structure B_S.

Let then $B = (G, \Theta)$ be the pair representing the graph G and the set of conditional independence $\Theta = \{\theta_1, \theta_2, \ldots, \theta_n\}$. We want to find the maximum a posterior (MAP) probability of the pair B given the data D Hankin et al. (2010),

$$\begin{aligned} B &= \arg\max_{B} P(B|D) \\ &= \arg\max_{B} P(D|B)P(B) \\ &= \arg\max_{G,\Theta} P(D|G,\Theta)P(\Theta|G)P(G) \end{aligned} \tag{S5}$$

and we call $P(D|G,\Theta)$ the likelihood of the data D given the pair $B = (G, \Theta)$. From the Bayes Theorem

$$P(D|G) = \int_{\Theta} P(D|G,\Theta)P(\Theta|G)d\Theta \tag{S6}$$

is the marginal likelihood. Assuming $P(\Theta|G)$ is a Dirichlet distribution (Hankin et al., 2010) (since it is the conjugate prior distribution of the categorical distribution and multinomial distribution (the distribution over observed counts of each possible category in a set of categorically distributed observations)), Heckerman (1994) defined The *Bayesian-Dirichlet* score, BD :

$$p(D|G) = \prod_{i=1}^{n}\prod_{j=1}^{q_i}\frac{\Gamma(N'_{ij})}{\Gamma(N'_{ij}+N_{ij})}\prod_{k=1}^{r_i}\frac{\Gamma(N'_{ijk}+N_{ijk})}{\Gamma(N'_{ijk})} \tag{S7}$$

in which n is the number of nodes, r_i is the number of states of the node i and q_i is the number of possible instantiations of the parents of the node i. $\Gamma(\cdot)$ is the Gamma function, N_{ijk} is the number of times x_i took the value k given the parent configuration j, $N_{ij} = \sum_{k=1}^{r_i} N_{ijk}$ and $N'_{ij} = \sum_{k=1}^{r_i} N'_{ijk}$. Since the quantisation level of the variables, r, is inside a product, it is clear that the learning algorithm can become very expensive if the quantisation is high enough. Nonetheless, if the quantisation is too low, the discrete dataset will be lost its properties and change behaviour, leading to an incorrect DAG to be assumed as the best BN to fit the original data.

The BD function when in the position of the scoring function to find the best BN is called *Bayesian Dirichlet equivalence with uniform prior metric, BDeu*. BDeu scores equally DAGs that entail the same conditional independencies, i.e., are Markov-equivalent structures (Bielza and Larranaga, 2014). There are other scoring methods like K2 (Behjati and Beigy, 2020), the Bayesian Information Criteria (BIC) (Bhat and Kumar, 2010) or a simple maximum likelihood estimation (Ben-Gal, 2008).

Inference in a BN is the process of obtaining the Conditional Probability Distribution (Neapolitan et al., 2004; Heckerman and Wellman, 1995) from the joint distribution. This process is achieved after having the BN structure, such that all the dependencies between the variables are established. Since the joint distribution, P is given by

$$P(x_1, x_2, \ldots, x_n) = \prod_{x\in\{X\}} p(x|\text{pa}_x), \tag{S8}$$

it is clear that both the computational complexity and the memory consumption of the exact inference algorithm depends on the product of the number of parents of each variable and its quantisation level. Since it can soon become unfeasible, some approximate algorithms were developed, e.g. Pearl's message-passing algorithm (Murphy, 1999; Pearl, 1994) and some special cases of DAGs like trees have a simple method to find the inference as well (Neapolitan et al., 2004; Huang and Darwiche, 1996). In this text, the selected inference algorithm is called *Variable Elimination* (Butz et al., 2009) and exploits dependencies in the structure to marginalise some variables, in practice eliminating them from the computation. However, Cooper has demonstrated that all know inference algorithms in BN are NP-hard (Cooper, 1990). In this paper, the discretisation used to address this problem was a variant of the adaptive discretisation proposed by Darbellay and Vajda (1999).

Markov property and stationarity

Let X_t be a stochastic process and let $F(X)$ be its cumulative distribution function. X_t is stationary (or strongly stationary) (Park and Park, 2018) if

$$F(x_{t_1}, x_{t_2}, \ldots, x_{t_n}) = F(x_{t_1+\tau}, x_{t_2+\tau}, \ldots, x_{t_n+\tau})\,\forall\,\tau\in\mathbb{R}, \forall\,t_n, n\in\mathbb{N}, \tag{S9}$$

in which the distribution F is invariant to time translations. In practice this imply that all the infinite moments of the process X_t were needed to fully decide if it is stationary. For practical purposes its

common to talk about *wide-sense stationarity* (WSS) or weak stationarity when talking about real time-series. In this case, a stochastic process is said to be WSS-stationary or simple stationary if its first moment $m_X(t)$ is time invariant, i.e. $m_X(t) = m_X(t+\tau)\,\forall\,\tau \in \mathbb{R}$ as well as its autocovariance $K_{XX}(t_1,t_2) = \mathbf{E}[(X_{t_1}-\mu_1)(X_{t_2}-\mu_2)]$, and its variance (second moment $E[(X_t)^2]$) is finite and constant in different time-slices).

Let $\{\mathbf{X_t}\}$ be a strictly stationary d-dimensional time-series process; it follows the Markov Property (MP) if $P(X_{t+1}|X_t, X_{t-1}, X_{t-2}, \ldots, X_0) = P(X_{t+1}|X_t)$ [1]. Let $I_t = \{X_t, X_{t-1}, \ldots, X_0\}$ be the information set corresponding to the past and present of a time-series; the hypothesis test of interest would be $H_0 : P(X_{t+1} \leq \mathbf{x}|I_t) = P(X_{t+1}|X_t)\,\forall\,\mathbf{x} \in \mathbb{R}^d$. Under H_0 the vector I_{t-1} contains redundant information about the future $t+1$. Alternatively $H_1 : P(X_{t+1} \leq \mathbf{x}|I_t) = P(X_{t+1}|X_t)$. There are a series o non-parametric tests for the MP; using the Chapman-Kolmogorov Equation we can write

$$g(\mathbf{X_{t+1}}|\mathbf{X_{t-1}}) = \int_{\mathbb{R}^d} g(\mathbf{X_{t+1}}|\mathbf{X_t}=\mathbf{x})g(\mathbf{X_t}=\mathbf{x}|\mathbf{X_{t-1}})d\mathbf{x} \tag{S10}$$

The MP is an important statistical tool to indicate how hard a time series can be to predict. First of all, by definition, non-stationary time-series do not obey the MP and if they lie in the hardest classes of non-stationarity such as piecewise stationarity and arbitrary variation they are hard (or even impossible) to predict. The duration of the time window is also important since some stationary or cyclostationary functions can be defined as non-stationary or unpredictable inside a short interval of its duration. But the most important conceptual information provided by the MP is how the past affects the future of a time series and can be a good explanation for the difficulty of predicting some functions.

DBN

Dynamic Bayesian Networks (DBN) are Bayesian Networks in which there is a time-dependency over the distributions (Ghahramani, 1997). There are simple examples of DBNs such as Kalman Filters (Chen et al., 2003) and Hidden Markov Models (Russell and Norvig, 2002).

DBNs were first introduced by Dagum et al. (1992) as a way of dealing with multivariate time series. The idea was to go over some classification models with the BN to a forecast model of those time series. To achieve this, Dagum first defined the *basal network* as the interaction between the variables inside a time-slice (stationarity assumption). When there is no update on the belief network, the basal model can be extended to several continuous time-slices (until it becomes obsolete and loses adherence).

However, for more complex real-world multivariate time-series data, it often happens that the basal network does not exist since the dependencies between the variables change over time-slices. In that case, it is needed to determine how many time-slices are necessary for forecast; it could also be the case that some variables become completely disconnected from the network.

REFERENCES

Behjati, S. and Beigy, H. (2020). Improved k2 algorithm for bayesian network structure learning. *Engineering Applications of Artificial Intelligence*, 91:103617.

Ben-Gal, I. (2008). Bayesian networks. *Encyclopedia of statistics in quality and reliability*, 1.

[1] This definitions is following what is called a first order Markov process; for generalisation, a k-th order Markov process follows the Markov Property $P(X_{t+1}|X_t, X_{t-1}, X_{t-2}, \ldots, X_0) = P(X_{t+1}|X_t, X_{t-1}, \ldots, X_{n-k+1})$.

Bhat, H. S. and Kumar, N. (2010). On the derivation of the bayesian information criterion. *School of Natural Sciences, University of California*, 99.

Bielza, C. and Larranaga, P. (2014). Bayesian networks in neuroscience: a survey. *Frontiers in computational neuroscience*, 8:131.

Butz, C. J., Chen, J., Konkel, K., and Lingras, P. (2009). A formal comparison of variable elimination and arc reversal in bayesian network inference. *Intelligent Decision Technologies*, 3(3):173–180.

Cai, H. (2017). Assessing and modeling community resilience to coastal hazards using a bayesian network. *Annals of the American Association of Geographers*.

Chaturvedi, I., Ragusa, E., Gastaldo, P., Zunino, R., and Cambria, E. (2018). Bayesian network based extreme learning machine for subjectivity detection. *Journal of The Franklin Institute*, 355(4):1780–1797.

Chen, Z. et al. (2003). Bayesian filtering: From kalman filters to particle filters, and beyond. *Statistics*, 182(1):1–69.

Contaldi, C., Vafaee, F., and Nelson, P. C. (2019). Bayesian network hybrid learning using an elite-guided genetic algorithm. *Artificial Intelligence Review*, 52(1):245–272.

Cooper, G. F. (1990). The computational complexity of probabilistic inference using bayesian belief networks. *Artificial intelligence*, 42(2-3):393–405.

Cooper, G. F. and Herskovits, E. (1992). A bayesian method for the induction of probabilistic networks from data. *Machine learning*, 9(4):309–347.

Dagum, P., Galper, A., and Horvitz, E. (1992). Dynamic network models for forecasting. In *Uncertainty in artificial intelligence*, pages 41–48. Elsevier.

Darbellay, G. A. and Vajda, I. (1999). Estimation of the information by an adaptive partitioning of the observation space. *IEEE Transactions on Information Theory*, 45(4):1315–1321.

Ghahramani, Z. (1997). Learning dynamic bayesian networks. In *International School on Neural Networks, Initiated by IIASS and EMFCSC*, pages 168–197. Springer.

Hankin, R. K. et al. (2010). A generalization of the dirichlet distribution. *Journal of Statistical Software*, 33(11):1–18.

Heckerman, D. (1994). Learning bayesian networks: The combination of knowledge and statical data. *Proceedings of Uncertainty in Artificial Intelligence, 1994*.

Heckerman, D. and Wellman, M. P. (1995). Bayesian networks. *Communications of the ACM*, 38(3):27–31.

Huang, C. and Darwiche, A. (1996). Inference in belief networks: A procedural guide. *International journal of approximate reasoning*, 15(3):225–263.

Jiang, W., Cao, Y., and Deng, X. (2019). A novel z-network model based on bayesian network and z-number. *IEEE Transactions on Fuzzy Systems*, 28(8):1585–1599.

Kouziokas, G. N. (2020). A new w-svm kernel combining pso-neural network transformed vector and bayesian optimized svm in gdp forecasting. *Engineering Applications of Artificial Intelligence*, 92:103650.

Larranaga, P., Poza, M., Yurramendi, Y., Murga, R. H., and Kuijpers, C. M. H. (1996). Structure learning of bayesian networks by genetic algorithms: A performance analysis of control parameters. *IEEE transactions on pattern analysis and machine intelligence*, 18(9):912–926.

Lee, S.-h., Kang, J. E., Park, C. S., Yoon, D., and Yoon, S. (2020). Multi-risk assessment of heat waves under intensifying climate change using bayesian networks. *International Journal of Disaster Risk Reduction*, 50:101704.

McCann, R. K., Marcot, B. G., and Ellis, R. (2006). Bayesian belief networks: applications in ecology and natural resource management. *Canadian Journal of Forest Research*, 36(12):3053–3062.

Moe, S. J., Haande, S., and Couture, R.-M. (2016). Climate change, cyanobacteria blooms and ecological status of lakes: a bayesian network approach. *Ecological Modelling*, 337:330–347.

Murphy, K. (1999). Pearl's algorithm and multiplexer nodes. Technical report, Citeseer.

Nasereddin, H. H. and Omari, A. A. R. (2017). Classification techniques for automatic speech recognition (asr) algorithms used with real time speech translation. In *2017 Computing Conference*, pages 200–207. IEEE.

Neapolitan, R. E. et al. (2004). *Learning bayesian networks*, volume 38. Pearson Prentice Hall Upper Saddle River, NJ.

Park, K. I. and Park (2018). *Fundamentals of Probability and Stochastic Processes with Applications to Communications*. Springer.

Pearl, J. (1994). Belief networks revisited. *Artificial intelligence in perspective*, pages 49–56.

Robinson, R. W. (1977). Counting unlabeled acyclic digraphs. In *Combinatorial mathematics V*, pages 28–43. Springer.

Russell, S. and Norvig, P. (2002). Artificial intelligence: a modern approach.

Stanley, R. P. (1973). Acyclic orientations of graphs. *Discrete Mathematics*, 5(2):171–178.

Tsamardinos, I., Brown, L. E., and Aliferis, C. F. (2006). The max-min hill-climbing bayesian network structure learning algorithm. *Machine learning*, 65(1):31–78.

Zweig, G. (2003). Bayesian network structures and inference techniques for automatic speech recognition. *Computer Speech & Language*, 17(2-3):173–193.

Zweig, G. and Russell, S. (1998). Speech recognition with dynamic bayesian networks. In *Proceedings of American Association for Artificial Intelligence (1998)*. University of California, Berkeley.
